# Analysis of atomic beam collimation by laser cooling

**DOI:** 10.1038/s41598-018-28218-y

**Published:** 2018-07-02

**Authors:** Shangyan Li, Min Zhou, Xinye Xu

**Affiliations:** State Key Laboratory of Precision Spectroscopy, East China Normal University, Shanghai, 200062 China

## Abstract

The collimation of a thermal atomic ytterbium beam utilizing a two-dimensional optical molasses is analysed by employing the Monte Carlo simulation. The dependencies of the collimation efficiency on power, frequency detuning and beam size of the laser are studied for various conditions, especially for the case of an imbalanced laser intensity and an impure laser polarization. The influences of these imperfect factors are discussed, and the lowest transverse temperature by the collimation in the experiment is evaluated.

## Introduction

As is well known, the collimation of the atomic beam becomes an important stage in the experiments on atom optics^1–4^, such as atomic clocks^5–10^, atomic interferometers^11,12^, and atomic lithography^13,14^. Besides mechanical ways, numerous techniques of manipulating the position and velocity of atoms by using the laser light have been demonstrated for beam collimation. The first demonstration employed a red detuned light overlapped the atomic beam with a radial intensity gradient for generating the transverse dipole forces^15^. Apart from dipole forces, scattering forces can collimate the atomic beam as well. Atomic beam deceleration by laser-radiation pressure was first reported in ref.^16^. Later V. I. Balykin’s team demonstrated a radiative collimation experiment through two dimensional (2-D) cooling of atoms by laser-radiation pressure in an axisymmetric light field formed by a reflecting axicon^17^. A technique called atomic lens has appeared with the development of the laser cooling methods. It uses an intense standing wave formed by blue-detuned lasers and does not saturate at high intensity^18^. It is noted that, the radiative collimation only works well under low light intensity and the atomic lens methods are not suitable for experiments with limited laser power.

A method called optical molasses finally becomes widely adopted in the atomic collimation, which can eliminate the compromise between the light intensity limit and the collimation efficiency^19^. The classical optical molasses took advantage of the laser-radiation pressure. It was improved later with the Lin⊥Lin configuration, which includes two counter-propagating light waves with orthogonal linear polarizations. This special light field, in which the light-shifted energies oscillate in space with a period of $\lambda /2$, forces atoms to be more likely at the uphill state than the downhill state. The atoms lose energy when propagating through the light field and “climbing” the potential hill. An alternative approach is to use the ${\sigma }^{+}-{\sigma }^{-}$ configuration. In this case the two counter-propagating waves are absorbed with different efficiencies, which gives rise to unbalanced radiation pressures. These two cooling methods can reach the level of sub-Doppler temperatures^20^.

It is always necessary to calculate the collimation efficiency whether one designs the cold-atom systems or optimizes the experiments. Although the theoretical model of the optical molasses for the atomic cooling has been widely studied and accepted, the collimation process seems more complex. Direct calculation of the collimation efficiency is very difficult and not suitable for systems with multiple stochastic processes^2^. The existing research in the field of the 2-D atomic beam collimation is not yet complete. For example, the cooling forces are regarded as damping forces and are characterized as near-linear with the velocity of the atoms^3^. In fact, the transverse velocity of an atomic beam depends on the oven temperature and the structure of the pre-collimator, which may lead to a deviation from the linear approximation. The Sisyphus effect is taken into consideration, while the effect of the polarization purity is not. Some researches discuss the influence of the imbalanced light intensity, but further quantitative study is necessary because the intensity imbalance is unavoidable in an experiment^19^.

The most widely used scheme of atomic beam collimation is still based on the optical molasses method^3,4,14^. Monte Carlo (MCL) simulation method which has been well-established is widely used in the field of cold atom physics^18,21,22^ and can be used for this research. We use MCL methods to simulate the process of collimating a thermal ytterbium (Yb) beam with the 2-D optical molasses. Firstly, the dependences of the collimation efficiency on power, frequency detuning, beam size of the laser and spout size of the oven in ideal conditions are studied. Then the influences of some non-ideal factors, especially for cases with an unsymmetrical laser intensity and an impure laser polarization are investigated quantitatively. We also compare the Lin⊥Lin and ${\sigma }^{+}-{\sigma }^{-}$ configurations, followed by a discussion of the dominating effects of the beam collimation. Finally, we draw some conclusions about the design and optimization of the 2-D atomic beam collimation.

## Analysis Under Ideal Conditions

### Initial state

First of all, we define the ideal conditions that remain constant in each simulation. Then, the method we choose for collimating the atomic beam is Doppler cooling (DC), and the dominant cooling force is the radiative force. Besides, the interaction between atoms is not taken into account. The classic setup of the 1-D atomic beam collimation consists of two symmetric lasers that are entirely identical in their characteristics, such as the laser power, the detuning, and the spot size in this part of the paper. In order to collimate the atomic beam in two dimensions, two sets of 1-D setups are required. One is employed in the horizontal collimation and the other one is used for the vertical collimation. Table 1 lists the default simulation values of the factors that have a significant influence on the initial conditions of the system.

**Table 1:** Default values of some variables in the collimation simulation.

Simulation variable	Default value
Critical divergence angle (mrad)	15
Diameter of oven spout (mm)	3
Oven temperature (K)	673
Laser power (mW)	10
Detuning (MHz)	−5

By calculating the distribution of the atomic divergence angle, we find that almost all the atoms which shoot out with a large critical divergence angle ${\theta }_{i}$ cannot reach the collimation field, thus being dissipated. To reduce the spout wastage to 2.5%, a pre-collimator with ${\theta }_{0}$ < 15 mrad should be designed. For gaseous ytterbium, the thermal motion level depends on the temperature. The velocity of Yb atoms in the oven obeys the Maxwell-Boltzmann distribution, while the velocity of atoms that spout out of the oven nozzle becomes the modified Maxwell-Boltzmann distribution. When the laser intensity and detuning are set, the damping ratio $\beta$ is obtained. It should be mentioned that the laser power stands for the power in one dimension, so the total power is doubled.

The criterion we use to measure the laser collimation effect is the equivalent transverse temperature in the atomic beam. The temperature depends on the average atomic kinetic energy $\bar{E}$ in the following way: $T=2\bar{E}/{k}_{B}$, where ${k}_{B}$ is the Boltzmann constant.

### Monte carlo simulation of the 2-D laser collimation

We use the initial state to build an atomic beam model and program a random number generator which is used in emulational sampling of the original atomic factors. Then the force of the collimation laser is calculated. In this section, only the DC force is taken into consideration, and the effects of the Sisyphus cooling and the polarization gradient cooling (PGC) will be discussed in the next section.

**Figure 1:**
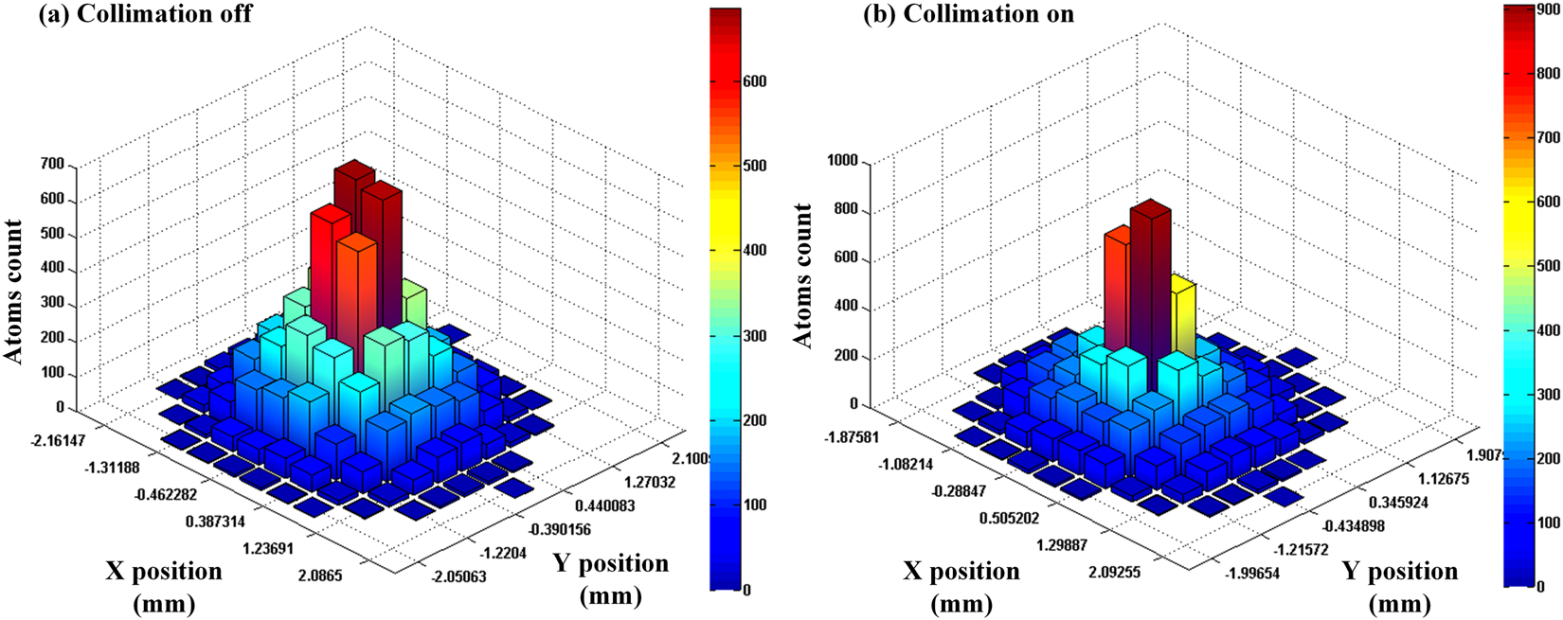
The position distribution of the atomic beam obtained from the MCL simulation. The deceleration length is 30 mm and there are 10,000 emulational atoms. Other parameters are set to be default.

The DC force is an atomic velocity dependent function^23^, so we use the Runge-Kutta method to solve the atomic trajectories and velocities in the laser field. Figure 1 shows the atomic position distribution in the cross section. We can see the difference between collimation OFF in Fig. 1(a) and ON in Fig. 1(b). When the cooling lasers take effect in the collimation, the atomic beam diameter decreases, which essentially is the desired effect. However, the atomic beam is not concentrated enough with the default collimation factors listed in Table 1, so further analysis and optimization are necessary.

### Analysis of light spot size

The collimation area, which is associated with the light spot size, has an important implication for the cooling time. The laser with a larger spot size can collimate more atoms within a longer time. However, the cost is the decrease in the light intensity. In the following we search for an optimal range of the spot size.

**Figure 2:**
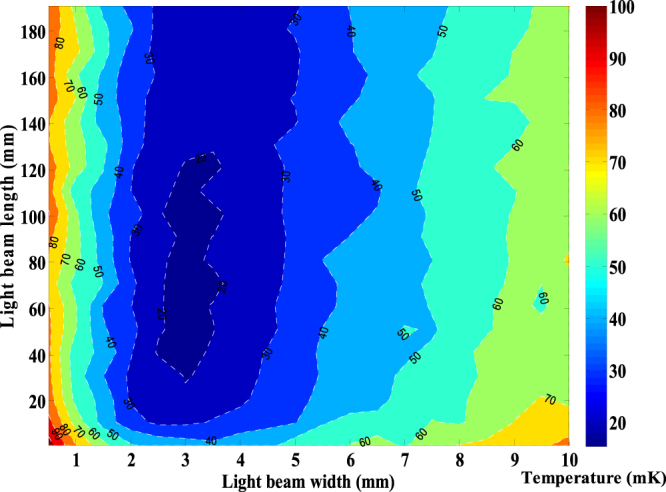
The transverse equivalent temperature of the atomic beam after being collimated by laser beams with different spot sizes. In this simulation, the efficient size range is marked with the dark blue colour. Even at the optimal size, the lowest equivalent temperature is still far above 0.7 mK, which is the Doppler cooling limit of the 399 nm transition in Yb.

**Figure 3:**
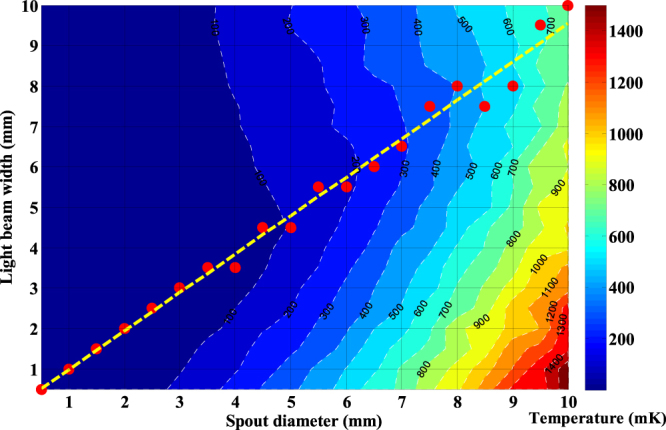
Influence of the oven spout diameter and the light spot width on the atomic beam collimation. The width at which the laser has the best collimation effect is marked by the red dots, and the yellow short dashed line is the linear fitting for the guide to eyes.

The oven temperature is set at 673 K, at which the most probable velocity of atoms is ${v}_{mp}=310\,{\rm{m}}/{\rm{s}}$. As seen in Fig. 2, the optimal light spot width is around 3 mm which is near the diameter of the oven spout. To confirm whether it is coincidental, we study the optimal light spot width with different spout diameters, as shown in Fig. 3. The linear fitting result shows that when the diameter of the oven spout is changed, the optimum light spot width changes linearly.

### Analysis of detuning optimization

Both the cooling laser detuning and the Doppler shift caused by the velocity of atoms contribute to the spontaneous force. In this section, the size of laser beam is 3 × 30 mm^2^ and the oven temperature is 673 K. We simulate the dependences of the transverse temperature on laser detuning for different laser powers. The laser beams are frequency shifted slightly below the atomic resonance. The red detuning is necessary to ensure the forces are opposite between the counter-propagating laser beams. However, if the detuning is too large, the cooling force will be decreased because the probability of spontaneous radiation becomes small. So there should be an optimum detuning.

**Figure 4:**
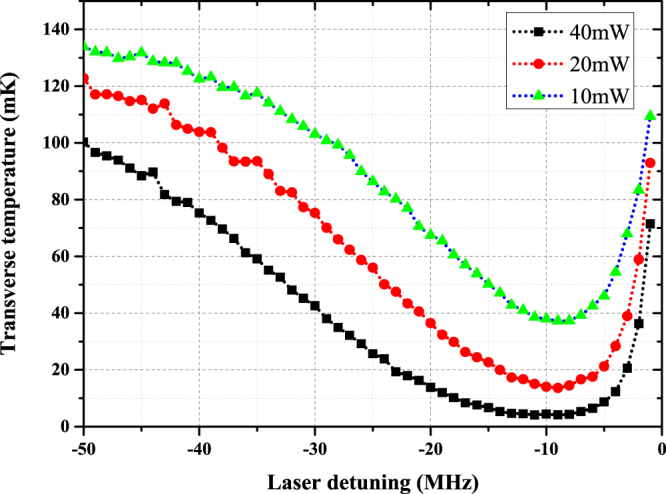
The dependence of the transverse temperature on the laser detuning. The black squares indicate the simulation results with 40 mW laser power. The red circles are the results in 20 mW and the green triangles are the results in 10 mW. The laser power is mean to the sum of the 2-D collimation lasers.

The optimum detuning is around −10 MHz for different laser powers, as shown in Fig. 4. It is interesting to see the curves become flatter near the optimum detuning when the laser power is increased. The optimal detuning ranges, where the difference between the transverse equivalent temperature and the lowest is below 5 mK, are listed in Table 2.

**Table 2:** Optimal detuning range for different collimation laser powers.

Total collimation laser power (mW)	Optimal detuning range (MHz)
10	−11~−7
20	−14~−6
40	−17~−5

The calculation delivers the information that the transverse temperature becomes more sensitive to the detuning at low laser powers. It is well understood because the cooling force becomes saturated at high laser intensity. Additionally, the laser power has little influence on the optimum detuning.

### Analysis of laser power optimization

In the previous section, we find the saturation of the laser power. In order to make full use of the laser power, the analysis of the laser power optimization must be done at the optimum laser detuning −10 MHz.

**Figure 5:**
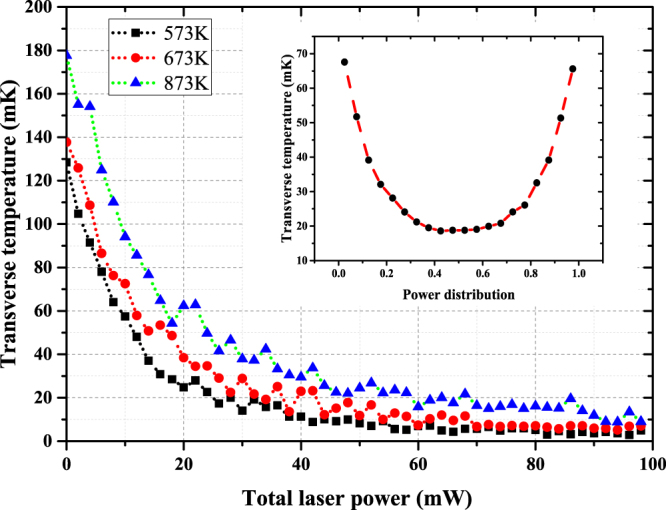
The dependence of the transverse temperature on the total laser power. The blue triangles are the results at 873 K oven temperature. The black squares are at 573 K, and the red circles stand for results at 673 K. The inset shows the dependence of the transverse temperature on the power distribution with a total laser power of 40 mW at 673 K. The power distribution, with its value lying in the range of zero to unity, represents the power ratio between lasers in the vertical and horizontal dimensions.

The oven temperature has evident influence on the initial transverse temperature. But the tendencies of the curves are similar, as shown in Fig. 5. The curves level off when the power is greater than 20 mW. If the polarizations of the counter-propagating laser beams are both linear and parallel, the red detuned standing wave will reduce the collimation effect.

## Analysis Under non-ideal Conditions

### Asymmetrical collimation laser power

The simulations with the ideal conditions help us find the optimum parameters and a narrow experimental optimizing range. But some non-ideal factors in the real experimental conditions may limit the collimation effect. We study the influence of intensity imbalance and polarization impurity of the lasers in order to provide the critical values of imperfect conditions.

A set of two counter-propagating laser beams are generally produced by one laser beam combined with a $0^\circ$ high reflector. However, the power loss of the reflector and the vacuum chamber windows cannot be ignored in a real experiment. This effect leads to the inequality of the counter-propagating lasers’ power that may influence the collimation effect.

We define the incident light power as ${P}_{i}$, and the reflected power as ${P}_{r}$. The transmissivity of two vacuum chamber windows are ${\gamma }_{1}$ and ${\gamma }_{2}$, respectively. The mirror loss is defined as ${\gamma }_{m}$. The reflected laser power can then be written as

1 $${P}_{r}={\gamma }_{1}{\gamma }_{2}{\gamma }_{m}{P}_{i}$$

**Figure 6:**
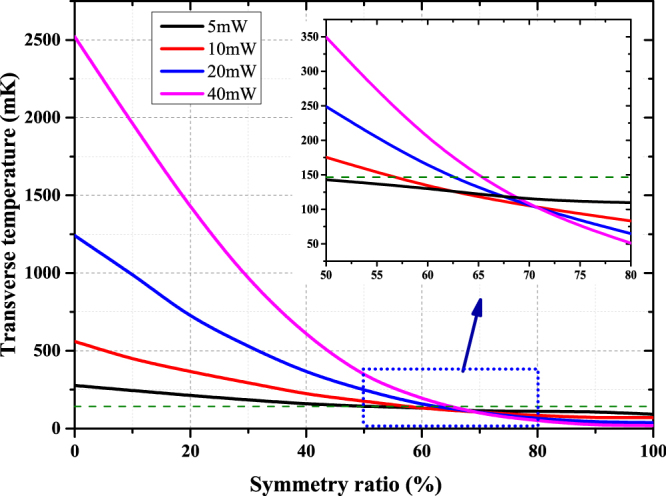
The dependence of the transverse temperature on the symmetry ratio. The green dashed line shows the transverse temperature without laser cooling. The solid lines represent the dependences of transverse temperatures on symmetry ratio at different collimation light powers. The inset shows an enlarged view with the symmetry ratio ranging from 50% to 80%.

At the location of the atomic beam, we have

2 $${P}_{+}={\gamma }_{1}{P}_{i}$$

and

3 $${P}_{-}={\gamma }_{2}{P}_{r}={\gamma }_{1}{\gamma }_{m}{\gamma }_{2}^{2}{P}_{i}$$

where ${P}_{+}$ is the power in the input direction and ${P}_{-}$ is the reflected one. The ratio of ${P}_{-}/{P}_{+}={\gamma }_{m}{\gamma }_{2}^{2}$ is defined as the symmetry ratio. We study the dependence of the transverse temperature on the symmetry ratio for various laser powers, as shown in Fig. 6. In addition, other simulation parameters are set to be optimal values.

As expected, the collimation effect by laser cooling is sensitive to the symmetry ratio. The intersections of the dashed line and solid lines indicate the critical values of symmetry ratio, below which the laser may heat or push away the atoms and the collimation gets weakened. Table 3 shows the critical values at different laser powers.

**Table 3:** Critical symmetry ratio at different laser powers.

Total collimation laser power (mW)	Critical symmetry ratio (%)
5	48
10	56
20	62
40	65

It should be noted that all the intersections of each two lines are nearly overlapped and are close to 70%, which means the dependence of the collimation effect on the laser power can be reversed. Furthermore, the transverse temperature seems independent of the laser power at this point. Fortunately, the optical loss of windows and mirrors is unlikely to cause such a poor symmetry ratio. Nevertheless, the calculation results indicate that the collimation will be more sensitive to the symmetry when the collimation laser power is higher.

### Impure polarization of the collimation laser

For a set of ideal conditions of orthogonal linear polarizations and equal amplitudes of both counter-propagating laser beams, one can write

4 $${{\bf{E}}}_{x}={E}_{0}\,\cos (\omega t-kz){{\boldsymbol{\varepsilon }}}_{x},$$

5 $${{\bf{E}}}_{y}={E^{\prime} }_{0}\,\cos (\omega t+kz){{\boldsymbol{\varepsilon }}}_{y},$$

so the total electric field

6 $${\bf{E}}(z,t)={E^{\prime} }_{0}\,\cos (\omega t+kz){{\boldsymbol{\varepsilon }}}_{y}+{E}_{0}\,\cos (\omega t-kz){{\boldsymbol{\varepsilon }}}_{x}.$$

As *E*_0_ = *E*′_0_, then

7 $${\bf{E}}(z,t)=\frac{{e}^{i(\omega t+kz)}+{e}^{-i(\omega t+kz)}}{2}{E}_{0}{{\boldsymbol{\varepsilon }}}_{y}+\frac{{e}^{i(\omega t-kz)}+{e}^{-i(\omega t-kz)}}{2}{E}_{0}{{\boldsymbol{\varepsilon }}}_{x}$$

8 $$\begin{array}{rcl}{\bf{E}}(z,t) & = & \frac{{E}_{0}}{2}({e}^{ikz}{{\boldsymbol{\varepsilon }}}_{y}+{e}^{-ikz}{{\boldsymbol{\varepsilon }}}_{x}){e}^{i\omega t}+\frac{{E}_{0}}{2}({e}^{-ikz}{{\boldsymbol{\varepsilon }}}_{y}+{e}^{ikz}{{\boldsymbol{\varepsilon }}}_{x}){e}^{-i\omega t}\\  & = & \frac{{E}_{0}}{\sqrt{2}}(\frac{{{\boldsymbol{\varepsilon }}}_{x}+{{\boldsymbol{\varepsilon }}}_{y}}{\sqrt{2}}\,\cos \,kz+i\frac{{{\boldsymbol{\varepsilon }}}_{y}-{{\boldsymbol{\varepsilon }}}_{x}}{\sqrt{2}}\,\sin \,kz){e}^{i\omega t}+\\  &  & \frac{{E}_{0}}{\sqrt{2}}(\frac{{{\boldsymbol{\varepsilon }}}_{x}+{{\boldsymbol{\varepsilon }}}_{y}}{\sqrt{2}}\,\cos \,kz-i\frac{{{\boldsymbol{\varepsilon }}}_{y}-{{\boldsymbol{\varepsilon }}}_{x}}{\sqrt{2}}\,\sin \,kz){e}^{-i\omega t}\end{array}$$

The first term of equation (8) is the negative-frequency component, while the second one is the positive-frequency component

9 $${\xi }^{+}(z)=\frac{{E}_{0}}{\sqrt{2}}(\cos \,kz\frac{{{\boldsymbol{\varepsilon }}}_{x}+{{\boldsymbol{\varepsilon }}}_{y}}{\sqrt{2}}-i\,\sin \,kz\frac{{{\boldsymbol{\varepsilon }}}_{y}-{{\boldsymbol{\varepsilon }}}_{x}}{\sqrt{2}})$$

${{\boldsymbol{\varepsilon }}}_{x}$ and ${{\boldsymbol{\varepsilon }}}_{y}$ are unit vectors whose directions are horizontal and vertical, respectively. The *z* axis is along the direction of laser lights. There are gradients of ellipticity when one moves along Oz. For the ^1^S_0_–_1_P_1_ transition in ^171^Yb (I = 1/2) shown inFig. 7, this kind of gradients can form a sub-Doppler cooling, which is usually called the Sisyphus cooling. And it can cool the atoms by dissipating the kinetic energy of atoms when atoms transit from one sublevel of the ground state to the excited state and go back to another ground sublevel in a finite time ${\tau }_{p}$. In addition, the detuning should be negative and the PGC is more powerful when the velocity of atoms is lower than the capture velocity ${\upsilon }_{c}$ which is related to the laser power. If the linearly polarized laser beams are contaminated by circular polarizations, the effect of PGC may be influenced and this is of interest to us.

**Figure 7:**
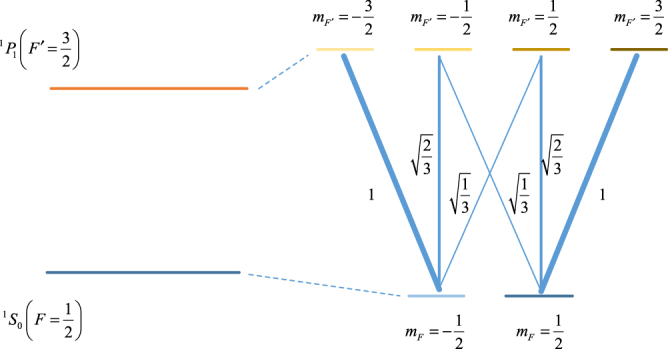
The atomic level scheme and Clebsh-Gordan coefficients for ^171^Yb ^1^S_0_ (F = 1/2)–^1^P_1_ (F′ = 3/2) transition at 399 nm.

The PGC force averaging over a wavelength could be written as

10 $$\bar{f}(\upsilon )=\frac{-\alpha \upsilon }{1+({\upsilon }^{2}/{\upsilon }_{c}^{2})},$$

where the friction coefficient is

11 $$\alpha =-\,3\hslash {k}^{2}\frac{\delta }{{\rm{\Gamma }}},$$

and $\alpha$ is independent of the laser power. However, the critical velocity ${\upsilon }_{c}$ is

12 $${\upsilon }_{c}=\frac{1}{2k{\tau }_{p}},$$

and it is proportional to the laser power. In addition, the calculation of equations (10–12) requires $k\upsilon \ll {\rm{\Gamma }}$.

If the laser polarizations form a complete Lin⊥Lin configuration, the optical pumping time ${\tau }_{p}$, which characterize the mean time that an atom takes to be transferred by a fluorescence cycle from one sublevel to another, is given by

13 $$\frac{1}{{\tau }_{p}}=\frac{{\rm{\Gamma }}}{9}\cdot \frac{{{\rm{\Omega }}}^{2}}{{\delta }^{2}+{{\rm{\Gamma }}}^{2}/4},$$

where the Rabi frequency ${\rm{\Omega }}$ is

14 $${\rm{\Omega }}=-\,\frac{d{E}_{0}}{\hslash },$$

where $d$ is the reduced dipole moment in the counter-propagating condition

15 $$d=\sqrt{\frac{3\pi \hslash {\varepsilon }_{0}{c}^{3}{\rm{\Gamma }}}{{\omega }^{3}}}.$$

When the linear polarization light is mixed with a fraction of circular polarization part, the energy shift between two sublevels of the ground state will be decreased, which leads to a weak PGC. We define the polarization purity ${\kappa }_{p}$ as

16 $${\kappa }_{p}=\frac{{P}_{linear}}{P},$$

where ${P}_{linear}$ is the power of the linear polarization component.

The expression of the cooling force with impure polarization is then

17 $${\bar{F}}_{c}={F}_{+}+{F}_{-}+\frac{-\alpha {\kappa }_{p}\upsilon }{1+({\upsilon }^{2}/{\upsilon }_{c}^{2})}.$$

Another sub-Doppler cooling method includes a pair of ${\sigma }^{+}$ and ${\sigma }^{-}$ polarized laser beams. In the Lin⊥Lin configuration, if the polarization of laser light is not pure, it becomes a combination of Lin⊥Lin and ${\sigma }^{+}-{\sigma }^{-}$ configurations. Nevertheless, DC still dominates the cooling process in the ${\sigma }^{+}-{\sigma }^{-}$ configuration, as ${J}_{g}=1/2$ for ^171^Yb.

Equation (17) is used to calculate the trajectory of atoms in the laser field, and the MCL simulation result can show the dependence of the transverse temperature of the atomic beam on the polarization purity.

**Figure 8:**
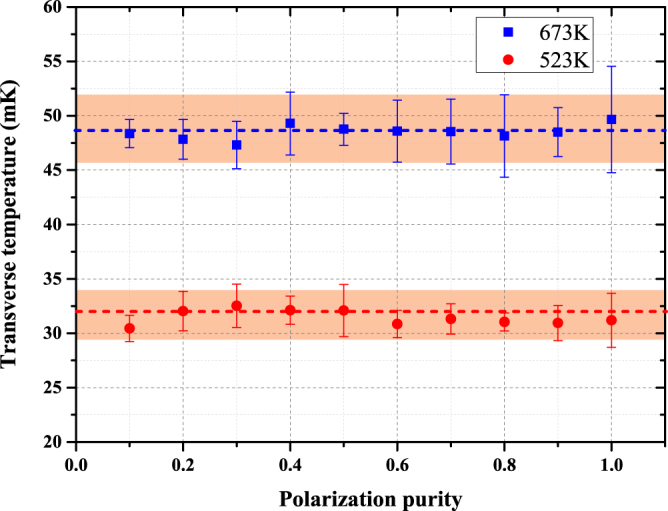
Dependence of the transverse temperature of the atomic beam on the polarization purity of the laser beam. Each point is averaged over 10 calculations to reduce the uncertainty. The blue squares correspond to results with a 673 K oven temperature. The red circles indicate the results with a lower oven temperature of 523 K. The orange shaded areas are the root mean square of the 10 times simulation results.

As shown in Fig. 8, we calculate the curve for the oven temperature at 673 K. The blue squares are the mean values of 10 MCL simulations, and the error bars are the statistic uncertainties. We cannot find an obvious influence of the polarization purity according to this curve. The reason that the sub-Doppler cooling is invalid may be because the capture velocity band is narrow. The laser power is 10 mW which corresponds to ${\upsilon }_{c}=0{\rm{.0436}}\,{\rm{m}}/{\rm{s}}$. Therefore, the transverse velocity of the atomic beam has been damped, and there are only a few atoms captured by the polarization gradient force.

**Figure 9:**
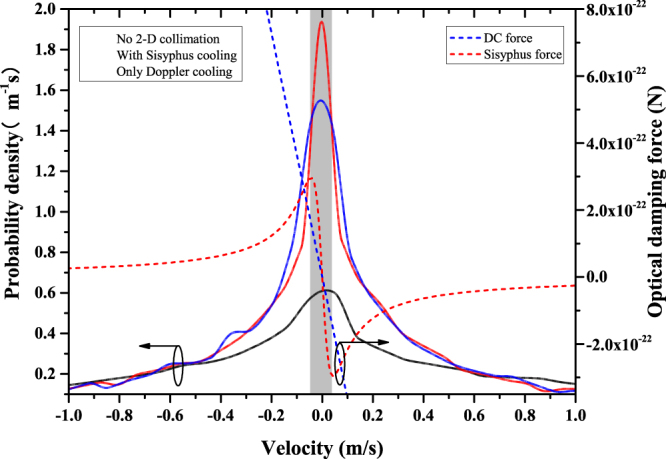
The solid curves are the probability densities as a function of the atomic transverse velocity acquired by the MCL simulation. The dashed lines are the damping forces in DC and PGC, respectively. The gray shaded area indicates the range of the capture velocity of the Sisyphus force.

Then we decide to increase the number of captured atoms. If we increase the laser power, the collimation force will be changed as well, which will further complicate the discussion. So we reduce the oven temperature to 523 K that has a narrower velocity distribution and more atoms may be captured by the polarization gradient force. Finally, the influence of polarization is still too weak on the transverse temperature, but significant on the velocity distribution as shown in Fig. 9.

The simulation conditions are optimized, and the number of the simulation atoms is $4\times {10}^{4}$. We notice that the capture velocity in DC should match the distribution of the non-collimation atomic transverse velocity. As a result, the atomic transverse velocity distribution is rearranged and most atoms are collimated by the DC force. However, the critical range of the Sisyphus force is much narrower. So it only interacts with a small part of the atomic beam and decelerate it. That is why the transverse temperature hardly changed with polarization purity.

The atomic beam collimation by laser cooling is used to decrease the divergence angle and increase the beam flux density in the preparation of cold atoms or the atomic lithography technology^3,24^. The polarization purity may be more meaningful when the critical velocity range is broadened or the atomic initial velocity distribution is more concentrated. But in our discussion, it is only a less important factor.

## Conclusion

The MCL method is an effective way to simulate the atomic dynamics. In this work, we investigate the influence of the laser power and the detuning on the atomic beam collimation. The lowest transverse temperature is 3 mK and is considerably hotter than the Doppler limit of 0.7 mK. The main reason is that there is insufficient interaction time. This algorithm can be reliably upgraded from only utilizing generalizations of ideal conditions to specific cases with non-ideal conditions. Then, it is shown that the polarization is not a dominant factor with our parameters, but the symmetry of the laser intensity has a remarkable influence on the collimation. Finally, the critical value of the intensity symmetry ratio is presented and we search for an explanation for that the collimation is insensitive to the light polarization. However, even using the optimal parameters and the sub-Doppler cooling method, the collimation by laser cooling does not reach the desired efficiency. In case the laser power is limited or the experimental setup has to be compact, this collimation unit is not necessary. This work will give a guideline for pursuing a good collimation of atomic beam in experiments on the optical lattice clocks with two-valence electron atoms.

## References

[1] Hänsch TW, Schawlow AL (1975). Cooling of gases by laser radiation. Opt. Commun..

[2] Joffe MA, Ketterle W, Martin A, Prichard DE (1993). Transverse cooling and deflection of an atomic beam inside a Zeeman slower. J. Opt. Soc. Am. B.

[3] Sukachev DD (2013). Collimation of a thulium atomic beam by two-dimensional optical molasses. Quantum Electron..

[4] Rathod KD, Singh PK, Natarajan V (2014). Cold beam of isotopically pure Yb atoms by deflection using 1D-optical molasses. Pramana - J. Phys..

[5] Akamatsu D (2014). Frequency ratio measurement of ^171^Yb and ^87^Sr optical lattice clocks. Opt. Express.

[6] Hinkley N (2013). An atomic clock with 10^–18^ instability. Science.

[7] Benjamin BJ (2014). An optical lattice clock with accuracy and stability at the 10^−18^ level. Nature.

[8] Poli N (2014). A transportable strontium optical lattice clock. Appl. Phys. B.

[9] Ushijima I, Takamoto M, Das M, Ohkubo T, Katori H (2015). Cryogenic optical lattice clocks. Nat. Photon..

[10] Koller SB (2017). Transportable optical lattice clock with 7×10^−17^ uncertainty. Phys. Rev. Lett..

[11] Mlynek J, Balykin V, Meystre P (1992). Optics and interferometry with atoms. Appl. Phys. B.

[12] Peters A, Chung KY, Chu S (2001). High-precision gravity measurements using atom interferometry. Metrologia.

[13] Timp G (1992). Using light as a lens for submicron, neutral-atom lithography. Phys. Rev. Lett..

[14] McGowan RW, Giltner DM, Lee SA (1995). Light force cooling, focusing, and nanometer-scale deposition of aluminum atoms. Opt. Lett..

[15] Bjorkholm JE, Freeman RR, Ashkin A, Pearson DB (1978). Observation of focusing of neutral atoms by the dipole forces of resonance-radiation pressure. Phys. Rev. Lett..

[16] Balykin, V. I., Letokhov, V. S. & Mishin, V. I. Cooling of sodium atoms by resonant laser emission. *Zh. Eksp. Teor. Fiz*. **78**, 1376–1385(1980) [*Soc. Phys. JETP* **51**, 692–696 (1980)].

[17] Balykin VI, Letokhov VS, Minogin VG, Rozhdestvensky YV, Sidorov AI (1985). Radiative collimation of atomic beams through two-dimensional cooling of atoms by laser-radiation pressure. J. Opt. Soc. Am. B.

[18] Aspect A, Dalibard J, Heidmann A, Salomon C, Cohen-Tannoudji C (1986). Cooling atoms with stimulated emission. Phys. Rev. Lett..

[19] Lett PD (1989). Optical molasses. J. Opt. Soc. Am. B.

[20] Dalibard J, Cohen-Tannoudji C (1989). Laser cooling below the Doppler limit by polarization gradients: simple theoretical models. J. Opt. Soc. Am. B.

[21] Chen YH, Tao HS, Yao DX, Liu WM (2012). Kondo metal and ferrimagnetic insulator on the triangular kagome lattice. Phys. Rev. Lett..

[22] Lu ST, Chen Y, Wu XH, Wang ZD, Li Y (2014). Three-dimensional sulfur/graphene multifunctional hybrid sponges for lithium-sulfur batteries with large areal mass loading. Sci. Rep..

[23] Balykin, V. I., Letokhov, V. S. & Sidorov, A. I. Radiative collimation of an atomic beam by two-dimensional cooling by a laser beam. *JETP Lett*. **40**, 1026–1029 (1984) [Pis’ma Zh. Eksp. Teor. Fiz. 40, 251–253 (1984)].

[24] McClelland JJ, Scholten RE, Palm EC, Celotta RJ (1993). Laser-focused atomic deposition. Science.

